# Patient Recruitment Characteristics for Wearable-Sensor-Based Outcome Assessment in Trauma Surgery

**DOI:** 10.3390/jcm14030805

**Published:** 2025-01-26

**Authors:** Benedikt J. Braun, Kira Hofmann, Chiara N. Meierhofer, Maximilian M. Menger, Tanja C. Maisenbacher, Carolina Vogel, Dannik Haas, Meir T. Marmor, Tina Histing, Eva-Marie Braun, The AO Smart Digital Solutions Task Force

**Affiliations:** 1 University Hospital Tuebingen, Eberhard-Karls-University Tuebingen, BG Klinik Tuebingen, 72076 Tuebingen, Germany; kira.hofmann@t-online.de (K.H.); c.meierhofer01@gmx.de (C.N.M.); mmenger@bgu-tuebingen.de (M.M.M.); tmaisenbacher@bgu-tuebingen.de (T.C.M.); cvogel@bgu-tuebingen.de (C.V.); dhaas@bgu-tuebingen.de (D.H.); thisting@bgu-tuebingen.de (T.H.); 2 AO Smart Digital Solutions Task Force, AO Foundation, 7270 Davos, Switzerland; meir.marmor@ucsf.edu; 3 Orthopaedic Trauma Institute (OTI), University of California, San Francisco General Hospital, San Francisco, CA 94110, USA; 4 Center for Integrative Oncology, Die Filderklinik, 70794 Filderstadt-Bonlanden, Germany; braun.em@web.de

**Keywords:** objective outcome, smartphone, clinical study

## Abstract

**Background/Objectives:** Using a patient’s own wearable sensor to obtain objective outcome data is a growing field in trauma allowing for the assessment of the recovery trajectory back toward the pre-injury performance. The aim of this study was to analyze recruitment characteristics and reasons for declined study participation in an orthopedic trauma study that measures postoperative recovery using wearables. **Methods**: Data from 225 patients screened for participation in a wearable-sensor-based outcome study were assessed. The influence of age, sex and injury location on study participation was analyzed. Reasons for patients declining to participate were investigated from the screening log. Availability and type of sensor system in patients agreeing to participate were analyzed. **Results**: Overall, 48% of patients agreed to participate. Age was the only significantly different factor between agreeing and declining patients (*p* < 0.05). The main reasons to decline study participation were technical difficulties with or inability to use the wearable device, lack of availability of a wearable, and general disinterest to participate in a study. Notably, 7% declined due to data safety concerns. **Conclusions**: The results show that age, availability of the wearable, and technical ability to use a wearable are the main obstacles impacting objective outcome measurement using a personal wearable device. In studies including geriatric patients, a dedicated device requiring no patient handling can be a valid option to improve enrollment. Understanding the reasons for declining to participate will facilitate the development of future sensor-based studies to address concerns of technical handling through alternative means of data harvesting and increase the inclusion rate. These outcomes will guide future study designs to optimize patient inclusion.

## 1. Introduction

Monitoring patient activity after trauma surgery with wearable sensors to analyze objective outcome data is a growing field in orthopedic trauma surgery. The current literature is still limited and lacking standardized inclusion protocols, outcome measures and techniques [1,2]. Currently two main measurement strategies exist: (i) using a preexisting wearable to measure patients’ postinjury performance (i.e., patient-owned smartphone or connected fitness watch—“bring-your-own-device” strategy) or (ii) using dedicated devices (i.e., smartwatches/bands) selected by the clinician/researcher which are then handed out to the patient [3,4]. While the dedicated device strategy offers advantages in interpatient comparability whilst obviating the need for device ownership by the patient, it suffers from the patients’ limited compliance using an unfamiliar digital device. Furthermore, dedicated devices limit data collection to prospective measurements from the timepoint of study inclusion onward.

The “bring-your-own-device” strategy employs devices already owned by patients, decreasing compliance and technology familiarity issues while making pre-injury data available for assessment [4,5]. This potentially allows for an individual recovery tracking back to a pre-injury functional level based on associated wearable metrics, most commonly the step count [6]. Limitations of this technique include comparability and availability of metrics between patients due to the multitude of devices employed, as well as data harvesting issues from different operating systems. However, using the “bring your own device” strategy offers several distinct advantages over the employment of a dedicated wearable device: it minimizes logistical and financial burdens, obviating the need to distribute, maintain, and retrieve a wearable device during study conduct and also clinical aftercare. In contrast to potentially unfamiliar dedicated devices that can have poor wear adherence, the use of personal devices guarantees that they blend in seamlessly with the patients’ daily lives, potentially improving long-term compliance [4,5]. Furthermore, by utilizing pre-injury data, this method offers a baseline comparison that is not possible with dedicated devices that are only used for postinjury deployment. This makes it possible to track functional recovery over time in relation to a patient’s typical, pre-injury activities, providing a personalized and clinically useful benchmark. The BYOD approach’s clinical applicability in trauma care is highlighted by recent research, showing that personal wearable technology can identify patients at risk of delayed recovery early in the postoperative course and predict functional recovery trajectories [6]. Despite the difficulties posed by device heterogeneity, improvements in accelerometry and data standardization methods should eventually lessen these problems and improve the dependability of cross-system comparisons. To maximize the potential effect of studies assessing outcome with wearables owned by patients, authors have recommended an extended analysis of device availability, system distribution, and reasons for declined study participation to better understand the patient population potentially assessable with wearable systems [1,5,6].

The aim of this study was to assess recruitment characteristics and device availability of a trauma cohort in a “bring-your-own-device” strategy research setting. Reasons for declined study participation and data delivery in a wearable-based orthopedic trauma study are compiled and analyzed to better understand future study setup for optimized patient inclusion.

## 2. Materials and Methods

The study in which the patients were approached for enrollment assesses a machine-learning models’ capability to predict functional recovery based on pre- and immediate postinjury patient activity as measured with wearable systems after orthopedic trauma as an observational cohort study. All patients above the age of 18 years with a pre-existing wearable sensor (smartphone or body-worn sensor) and any musculoskeletal mono-trauma of the upper or lower extremity were eligible for inclusion. Exclusion criteria included patients unable to provide informed consent, drug abuse (illegal drugs/the use of prescription or over-the-counter drugs for purposes other than those for which they are meant to be used or use in excessive amounts/alcohol dependency), pregnancy, and enrollment in another clinical study. Continuous patient screening was performed by two medical students. Patients were screened during their in-patient stay at a German level-1 trauma center. For the current analysis, only the screening log for study enrollment was assessed to determine recruitment characteristics and reasons for declined study participation and data delivery. Age, sex, and injury location were assessed for all patients and comparative statistics performed. Patients refusing to participate were asked for their primary reason to decline in free form. The reasons were than categorized for further, anonymous assessment. Statistical analysis and figure creation was performed with JASP (version 0.18.3; JASP, University of Amsterdam, Netherlands). Data were tested for normal distribution with the Shapiro–Wilk test, and accordingly, comparative statistics were performed with the Mann–Whitney U test or *t*-test. Contingency tables were tested with the Chi-Square test. *p* < 0.05 was defined as statistically significant. The study was approved by the responsible local ethics committee (Protocol code 790/2020BO2).

## 3. Results

**Figure 1:**
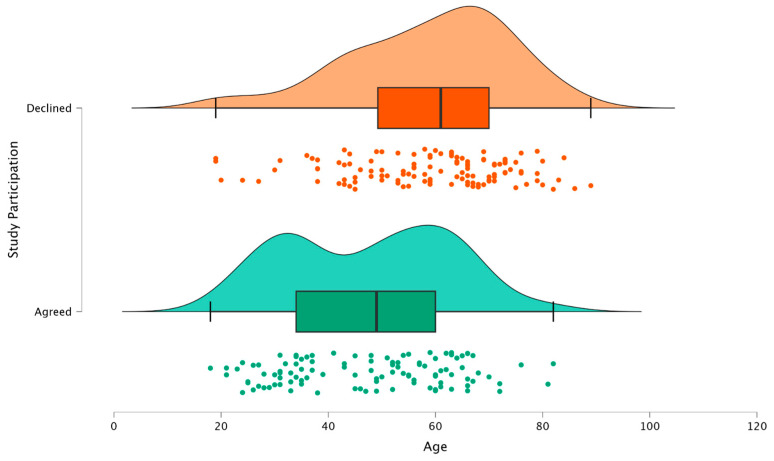
Raincloud plot visualizing patients agreeing (bottom, green) and declining (top, orange) to participate in the study. The *x*-axis shows the patient age in years. The point cloud shows individual age values for all patients in the group, and the boxplot shows median, upper, and lower quartile, as well as min/max whiskers. The top cloud graphs show the probability distribution.

Overall, 225 patients were screened for study participation. Of those, 107 (48%) agreed to participate using their own wearable device, whereas 118 declined. Comparing the groups of patients agreeing or declining to participate by age was the only significantly different factor (47.51 ± 15.48 vs. 58.98 ± 15.05 years; *p* < 0.05) (Figure 1).

**Figure 2:**
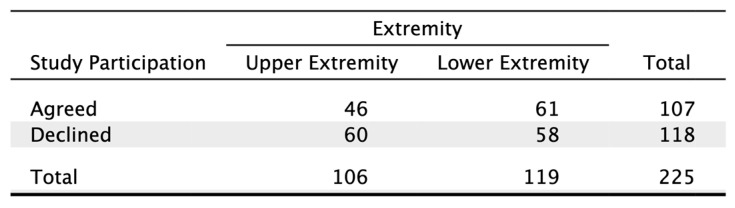
Contingency table showing study participation in relation to injury location.

**Figure 3:**
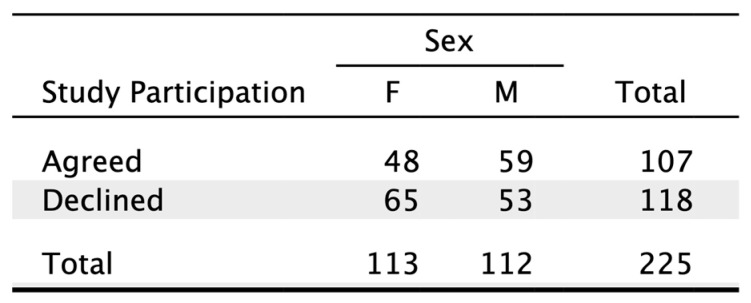
Contingency table showing study participation in relation to patient sex.

Neither sex (*p* = 0.127) nor injury location (*p* = 0.238) showed a significant influence on study participation (Figure 2 and Figure 3).

All patients declining to participate provided a primary reason to do so. These were grouped in 12 categories: Uninterested to participate in study, no smartphone/wearable available, technical difficulties to follow data transmission protocol, does not know how to use own smartphone/wearable, unable to understand German language informed consent, too many worries to focus on study, data safety concerns, feels too old to participate, use of smartphone too rare/does not carry smartphone enough, unable to use wearable due to injury, participation in another study, does not want to spend unnecessary time on phone. This can be further summarized into three potentially addressable categories during study construction: (1) No device available—addressable with dedicated devices; (2) missing technical skill—addressable by low-maintenance workflow/minimal input data harvesting; (3) skepticism towards data safety/phone use—addressable by patient education/data safety protocol (Table 1).

Of the included patients, the majority used devices based on the Apple Health Application (41/107, 38%). The second most common application was Samsung Health (28/107, 26%). Dedicated device applications through fitness trackers or pedometers (Fitbit, Polar, Garmin) were used by 16 patients (15%).

## 4. Discussion

Clinicians and researchers are increasingly relying on objective outcome data to tailor rehabilitation protocols, predict recovery trajectories, and make clinical and surgical decisions [7,8,9]. The use of wearable technology and mobile health sensors has given new opportunities to collect real-time data, enabling continuous monitoring of patient activity levels and functional outcomes outside the clinical setting [10,11,12,13]. This additional data offers a more nuanced understanding of patient recovery patterns in clinics and, as a potential study endpoint, facilitates the comparison of different surgical techniques, implant designs, and also therapeutic interventions. The majority of studies employing wearables in orthopedic trauma are set up as dedicated device studies, where all patients receive the same type of device. The study wearable is handed out at inclusion and prospectively collects patient data with the same device over the course of a study [1]. While this offers good interpatient comparability and uniform outcome measures, it is limited by patient wear compliance of a new system, as well as the lack of pre-injury data for recovery trajectory comparison [14,15]. As smartphones and personal fitness wearables are increasingly common in the general population, it may be possible to overcome the limitations of a dedicated device by using a patient’s pre-existing wearable device [5,6,16]. By using this strategy, compliance issues otherwise arising from a new wearable device are reduced while offering the ability to track the individual patient recovery based on pre-injury data collected by the device prior to injury and study inclusion [6].

In the context of our pre-study enrollment demographics, a significant disparity was observed in the age distribution between participants and patients declining to participate, with the latter group exhibiting a higher mean age, attributed to smartphone-related barriers (device availability and lack of technical skill). Despite the increased popularity of wearable technology within the general population, as well as clinical and research domains, an inverse relationship between age and the propensity to utilize smartphones and wearable devices has been documented [1,2,16,17,18]. This currently limits the applicability of the “bring-your-own-device” strategy in geriatric fracture populations. Researchers looking to set up a study aimed at geriatric patients should thus consider employing a dedicated device to ensure adequate recruitment within this group. However, data pertaining to the distribution of wearable technologies within the United States point to an increasing trend of using smartphones, tablets, and health apps also in a geriatric cohort [19]: Ownership percentages of smartphones, as reported by the United States Health Information National Trends Survey, exceeded 70% within the demographic of 56 to 65 years and approached 60% for individuals aged 66 to 75 years, only declining to below 40% in the demographic surpassing 75 years of age. Initiatives aimed at increasing engagement with wearable devices and enhancing smartphone literacy among the elderly have already been reported on with positive effect. Hence, potentially increasing enrollment rates will be observed in this demographic with the “bring-your-own-device” strategy in the near future [18].

The second most common barrier to study inclusion was a perceived lack of technical skill, which was associated with older patient age. This effect can be mitigated for both factors with the setup of study software, as well as through patient training and education through clinic and research staff. In fact, an easy-to-use data harvesting setup requiring only minimal input can mediate these issues. The majority of study participants in this cohort and others were Apple Health users, a program that easily and securely shares personal health and fitness data with their healthcare provider. However, to truly allow for a device-agnostic approach incorporating the many different systems used when employing the “bring-your-own-device” strategy, a cross-platform harvesting solution is needed. Different apps are currently on the market to facilitate this task among Android-based devices that can be tailored to the clinicians and researchers’ specific needs. Their applicability under the local data safety jurisdiction must be assured. However, depending on the study location and the current smartphone application market, individual data sharing through research assistants can be the only feasibly means of sharing [4,6,20]. The need for a device-agnostic approach to sensors and the data harvested from the devices in different fields is highlighted by other large scale research projects, such as the Mobilize-D consortium [21,22,23]. Currently, different development initiatives aimed at device-agnostic data harvesting in an EU- and US-data-safety-compliant fashion are underway, enabling an easy and safe means of data extraction without increased patient input in the near future. The need for this approach is highlighted by the multitude of systems used by patients in our cohort. Data harvesting solutions are currently in development to specifically collect medical wearable data, and local data safety regulations can address the patients’ concern about data safety. Interestingly, the rate of patients declining for data safety reasons was lower than expected at just over 5 percent. This might be attributable to our inclusion procedure and individual data harvesting approach that is based on two medical students acting as research coordinators, screening patients and ensuring individual data transfer directly with our study team. Until device-agnostic harvesting solutions with the purpose of collecting wearable data in a medical context become universally available, this personal and individual approach, while time consuming, is a feasible way to enroll patients and collect wearable data. This approach has been used in previous studies on the feasibility of the “bring-your-own-device” strategy [4,6].

Of note, despite the large variety of systems used by our patients, all smartphone and wearable users included were able to track their daily step count as a device-agnostic outcome parameter. This has been shown as one of the most relevant outcome metrics when considering objective patient physical activity status after orthopedic and orthopedic trauma procedures and in healthy adults of all ages [1,24,25,26]. The heterogeneity certainly raises questions concerning comparability of step counts and other outcome metrics between patients. This variety in the wearable technology and operating systems that patients use is certainly a major obstacle in research using the “bring-your-own-device” (BYOD) strategy. The precision and comparability of measures such as step count can be impacted by differences in hardware, software, and data processing between different devices. Regardless, this variation also mirrors actual use patterns, somewhat hinting at the findings’ generalizability. Furthermore, the BYOD strategy makes it possible to incorporate patient-specific pre-injury data, shifting the focus more towards individualized insights that specialized devices are unable to provide. Although cross-system disparities are still an issue, data standardization and device-independent analytic techniques are evolving rapidly. On the one hand, current research projects are focused on providing societal activity and step count norm data to provide a frame of reference [26]. On the other hand, emerging frameworks like the Mobilize-D consortium aim to standardize digital mobility outcomes in order to create more dependable and consistent measurements across various wearable platforms. The standardization of methods, as well as outcome parameters, could be used in future research to close gaps in inter-device comparability while maintaining the adaptability and patient-centered advantages of the BYOD approach.

**Figure 4:**
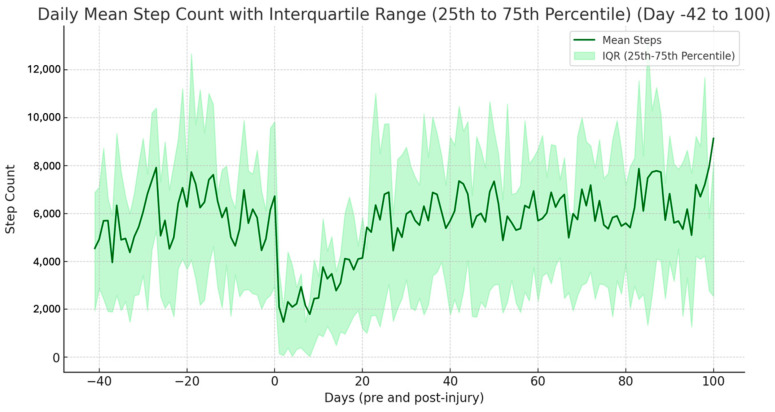
Exemplary visualization of the patient recovery process attainable with the bring-your-own-device approach from pre- to postinjury as tracked by the daily step count is shown in a trauma cohort [6]. Dark green shows the mean step count, light green the interquartile range. The X axis shows the days before (negative) and after (positive) injury, the Y axis the step count.

This undeniable limitation is thus also the major advantage of the approach in that it offers the reliability of comparing to the functional status of the same patient before an injury, something a dedicated device could not do. This allows for a continuous tracking of the patients functional recovery process from a pre-injury level throughout the post-injury course (Figure 4). While PROMs are able to accomplish this retrospectively, they are limited by recall bias [27]. The literature looking at the inter-device comparability of accelerometry data, as well as a growing body of reference data for common devices and wearable systems, will further address the challenges of comparing across different systems. Recent research underscores the importance of tracking physical activity through accelerometry measured daily step count as a key indicator of general health, as well as a frame of reference to determine the effect of interventions [26]: A population-based cross-sectional study established accelerometer-derived reference values for daily physical activity across different age and sex categories in Denmark, providing a first benchmark for further comparison. Participants in this study averaged 6095 steps per day and exhibited variations in cadence, sedentary time, and other metrics by age and sex. These findings emphasize the significance of establishing a larger set of musculoskeletal-injury-specific, population-level reference values, which could ultimately enhance clinical and research efforts to contextualize and evaluate patient activity levels during injury recovery in relation to “norm recovery trajectories”.

This study is limited by the geographic and societal specifics of the southern German region our trauma center is based in. Phone and wearable system distribution might differ in other regions and countries around the world. However, the general distribution of wearable systems, as well as operating systems, has been seen within these ranges in other studies out of the United States [4,28]. In addition, the phone and injury distribution might have been influenced by the specialized trauma population treated at our level-1 and trauma referral center. Furthermore, despite the inclusion of just over 200 patients this number is low considering the amount of trauma patients seen at our institution. This is due to the exploratory nature of our study and has to be confirmed in larger-scale studies on dedicated fracture entities in the future. The small sample size might not accurately represent the more general population of orthopedic trauma patients. Larger-scale studies with more varied cohorts are needed to validate and build upon our findings, even though this sample size was suitable for the exploratory character of the study. Additionally, medical students were involved in both patient recruiting and screening, raising the potential for selection bias. Even though we used standardized procedures to prevent bias, future research will incorporate a more varied screening staff to increase reproducibility and lessen the screening team’s potential influence.

## 5. Conclusions

The major obstacles to employing the “bring-your-own-device” sensor strategy to obtain objective study outcome data are patient age, device availability, and technical ability to use a wearable device. This limits the strategies broad applicability in a geriatric trauma setting, necessitating other measurement options. Other potential obstacles associated with data harvesting and data safety can be expected but addressed. Understanding the reasons of the patients declining participation can help construct future studies to address concerns of technical handling through alternative means of data harvesting to potentially increase inclusion rate.

## Figures and Tables

**Table 1:** This table shows the reasons to decline study participation, their relative frequency, and how the issues can be addressed.

Reason to Decline	Frequency (%)	Potentially Addressed By
Uninterested to participate in study	21	Continued patient education
No smartphone/wearable available	19	Switch to dedicated device
Technical difficulties to follow data transmission protocol	15	Low-maintenance workflow/minimal input data harvesting Continued patient education
Does not know how to use own smartphone/wearable	12	Switch to dedicated device Continued patient education
Unable to understand German language informed consent	8	Adaptation of study setup (i.e., translation service)
Too many worries to focus on study	7	Continued patient education
Data safety concerns	6	Patient education/data safety protocol
Use of smartphone too rare/does not carry phone enough	5	Continued patient education Switch to dedicated device
Unable to use wearable due to injury	3	Switch to dedicated device (outside zone of injury)
Feels too old to participate	2	Continued patient education Switch to dedicated device
Participation in another study	1	Adaptation of study setup (i.e., wearable study as adjunct)
Does not want to spent unnecessary time on phone	1	Low-maintenance workflow/minimal input data harvesting

## Data Availability

The original contributions presented in this study are included in the article. Further inquiries can be directed to the corresponding author.
